# The Effects of Acyl Chain Length on Antioxidant Efficacy of Mono- and Multi-Acylated Resveratrol: A Comparative Assessment

**DOI:** 10.3390/molecules27031001

**Published:** 2022-02-02

**Authors:** Han Peng, Fereidoon Shahidi

**Affiliations:** Department of Biochemistry, Memorial University of Newfoundland, St. John’s, NL A1C 5S7, Canada; hanp@mun.ca

**Keywords:** acylation, esterification, transesterification, lipophilicity, regioselectivity, antioxidant efficacy, bioavailability

## Abstract

Acylated derivatives of the dietary phenolic, resveratrol, were prepared via enzymatic and chemical transesterification modification with selected vinyl fatty acids to expand the potential application of resveratrol and its acylated derivatives in functional supplement, cosmetic/skincare, and pharmaceutical fields. The acylation was implemented using eight vinyl fatty acids with varying chain lengths (C2:0-C18:0). Eight monoesters enzymatically prepared, eight diesters and four triesters, chemically prepared, were isolated and purified and identified via MS (mass spectra) or/and NMR (nuclear magnetic resonance). The lipophilicity of resveratrol and its acylated derivatives was calculated using ALOGPS 2.1. Compared with related acylated products, resveratrol itself rendered higher antioxidant efficacy in all the antioxidant assays, namely DPPH, ABTS, FRAP, and ferrous chelation tests. Within various ester derivatives of resveratrol, short-chain fatty acid mono- and di-substituted resveratrols, especially the resveratrol monoacetate/diacetate, exhibited higher antioxidant efficacy in DPPH and ABTS assays than the rest of resveratrol derivatives, but the medium-chain monoesters of resveratrol, including caproate, caprylate, caprate, and laurate, showed a higher metal ion chelation ability compared to other acylated resveratrols. These results imply that resveratrol derivatives may be used in lipidic media as health-beneficial antioxidants.

## 1. Introduction

Resveratrol (3′,4′,5′-trihydroxystilbene) is a naturally occurring polyphenolic compound that functions as a phytoalexin of spermatophytes such as grapevine, mulberries, and peanuts. It is abundantly synthesized within the injured sites as a defence response against biotic and abiotic stresses [1]. Resveratrol is more commonly present in red wine rather than white wine, given that it is mainly distributed in grape skins and not the flesh [2]. Resveratrol has been extensively studied in both preclinical and clinical trials [3,4], and shows various bioactive properties and preventive effects on inflammation [5], type-II diabetes, obesity [6], cardiovascular disease [7], neurodegenerative disease [8], and cancer [9], among others.

Resveratrol with its myriad of bioactivities is a highly potent antioxidant. Resveratrol and its metabolites suppress pathophysiological indicators pertaining to lipid peroxidation, DNA modification/damage/lesion, and protein oxidation/misfolding/denaturation in vivo, by directly scavenging ROS or activating pathways that upregulate cells’ antioxidant defences [4,10,11]. Furthermore, resveratrol can interact with the respiratory chain in mitochondria as a respiratory chain inhibitor [12]. However, resveratrol’s poor pharmacokinetic results in vivo, particularly low bioavailability, low stability, and rapid metabolism, limit its commercial use as a nutraceutical, pharmaceutical, or functional food ingredient [13,14]. 

To date, various formulations and modification methods have been developed to efficiently deliver resveratrol to target tissues, including encapsulation, emulsification, and covalent modification, which may enhance chemical stability and absorption rate or/and retard the excretion/detoxification rate of resveratrol after oral administration [15,16]. Therefore, the preparation of novel resveratrol derivatives and improvement of their functional properties have been a recent research hotspot. Acylation of resveratrol has been used to increase resveratrol’s bioactivity and bioavailability [17,18]. For example, increased lipophilicity and consequentially improved permeability rate across the cellular membrane can be implemented by fully acylating resveratrol into resveratrol triacetate [19]. Furthermore, acylation may increase the oxidative stability and retard the rapid gastrointestinal degradation as well as in vivo metabolism and excretion of resveratrol that are partially contributed to by its hydroxylation, glucuronidation, and sulfation, because phase I/II metabolism are more difficult for acylated resveratrol due to the partially/fully ‘capped’ hydroxyl groups, which therefore exhibit an improved pharmacokinetic profile and more significant distribution in liver, spleen, heart, and lungs of rats compared to those of resveratrol itself [20]. 

Considering the different application fields, a specific/optimal acylation degree and acyl chain length of resveratrol should be noted. For example, inhibitory activity on PAF (platelet-activating factor), namely antithrombotic effect, of the resveratrol diacetate is much more potent than those of resveratrol and its mono- and tri-acetate derivatives [21]. In the human skin model, resveratrol butyrate and isobutyrate monoesters were biologically more active than their acetate, diacetate, and palmitate analogues in upregulating gene expression of SIRT 1, extracellular matrix proteins collagen, elastin, antioxidant enzymes, and various skin growth factors [22]. Furthermore, the length of the acyl chain also significantly biases the antioxidant efficacy of resveratrol derivatives. In a previous contribution from our laboratory, the antioxidant activities of 12 resveratrol monoesters acylated by using their corresponding fatty acid chlorides with varying chain length and degree of saturation/unsaturation (C3:0-C22:6) were compared. In this study, resveratrol mono-oleate and resveratrol monobutyrate performed best in DPPH (2,2-diphenyl-1-picrylhydrazyl) and ABTS [2,2′-azino-bis(3-ethylbenzothiazoline-6-sulfonic acid)] radical-scavenging assays, respectively [23]. This was attributed to the difference in their physical location in different solvent systems that was signified within the ‘polar paradox’ theory [24]. However, the influence of degree of acylation on antioxidant activity of resveratrol derivatives has not been verified so far. In the study reported here, the effects of both acyl chain length and acylation degree on antioxidant efficacy of resveratrol esters were investigated and analysed.

On the other hand, several chemical acylation mechanisms such as Mistunobu reaction and Steglich reaction or special acyl donors such as acyl chloride and acyl anhydride have been used to acylate phenolics, including resveratrol. However, natural phenolics possess multiple hydroxyl groups, and their backbone structures are usually unstable, thereby a lack of regioselectivity and considerable side reactions of chemical methods become their main disadvantages for the acylation of natural phenolics. Therefore, in recent years, the use of enzymatic processes for the synthesis of phenolic esters has become a promising alternative to chemical reactions. Enzymatic acylation can be conducted under milder reaction conditions with regioselectivities and enantioselectivities, particularly depending on a combination effect dictated by acyl donors, enzymes, temperature, pH, and solvents, among other factors. Lipases are widely recognized as suitable catalysts for esterification and transesterification as they are economical with high yield, stability, and accessibility. In this contribution, eight resveratrol monoesters, eight resveratrol diesters, and four resveratrol triesters were synthesized and purified to investigate the effects of degree of acylation and acyl chain length on antioxidant efficacy, including DPPH/ABTS scavenging, ferric reduction, and ferrous chelation abilities of resveratrol. The monoesters were prepared via one-step enzymatic transesterification using commercially available lipase (PS) and vinyl fatty acids (C2:0-C18:0). Resveratrol diesters and triesters were prepared via one-step base-catalysed transesterification using NaOH (sodium hydroxide) and vinyl fatty acids (C2:0-C18:0).

## 2. Results and Discussion

### 2.1. Preparation of Resveratrol Esters

Acylation alters the physicochemical and biological properties of compounds for the target application. The increased lipophilicity and stability of the resveratrol esters allowed them to be proposed as lipidic antioxidants used for cosmetics and skincare products or as main ingredients of topical/oral prodrugs, as mentioned before. More discussion of their application as antioxidants in the food and pharmaceutical field is provided in the analysis section of antioxidant efficacy.

In addition, although chemical acylation methods are generally not regioselective, they are still frequently employed to investigate the bioactivity difference between acylated resveratrol and its parent molecule. In this study, vinyl fatty acids with varying chain lengths (C2:0, C4:0, C6:0, C8:0, C10:0, C12:0, C14:0, C18:0) were employed to expand the application of resveratrol into oil-based food, nutraceuticals, and other daily chemical products. Lipase PS and NaOH served as catalysts for enzymatic and base-catalysed transesterification, respectively. Eventually, eight monoesters (resveratrol monoacetate/butyrate/caproate/caprylate/caprate/laurate/myristate/stearate) were prepared through enzymatic transesterification, and another eight diesters (resveratrol diacetate/butyrate/caproate/caprylate/caprate/laurate/myristate/stearate) and four triesters (resveratrol tributyrate/caproate/caprylate, stearate) were prepared through base-catalysed transesterification. The enzymatic reaction was only used for preparation of monoesters because the current enzyme (lipase PS) and reaction system (DMF, 50°, 12 h) mainly yielded monoesters (~70–90% of total ester products). Base-catalysed transesterification is much more efficient in synthesizing di-/tri-esters. During the reaction, the extent of acylation of crude reaction mixtures was monitored by TLC plates and confirmed by ESI-ToF-MS. The rough estimates of yields of ester products such as resveratrol laurates under current nonoptimized reaction conditions were around 9–15% (enzymatic transesterification) and 25–40% (base-catalysed transesterification) according to MS data. The yield of enzymatic transesterification in the current study was similar to the result (10.2 ± 1.3%) reported by Wang et al. [25], who also used the lipase PS for one-step enzymatic transesterification. In the same study, the yields under the same reaction condition but using lipases CSL, AKL, and CAL-B were 14.9, 4.6, and 9.4%, respectively [25]. Another study using lipases QLG or Novozym 435 gave yields of 75–80% monoacetate and 35–55% monostearate of resveratrol under reaction condition of 150–250 mM resveratrol, 2.25–3.75 M vinyl fatty acids, 150–229 mg/mL enzymes, and 5–6.55 mL 2-methyl-2-butanol for 12–160 h [26]. More optimized or nonoptimized reaction conditions along with their yields using different enzymes can be found in Table 1, and some of them show an excellent yield (more than 90%). Compared to the yields from other reports, the reaction condition and type of lipase used in the current studies need to be further optimized and adjusted, supposing that the future industrialized application is of interest.

### 2.2. Identification of Resveratrol Esters

**Figure 1:**
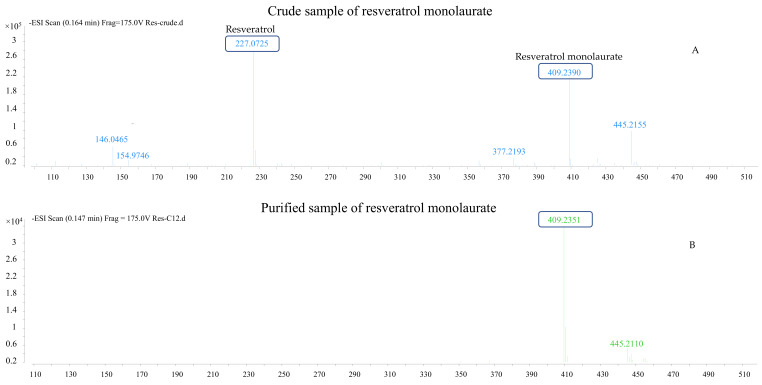
Mass spectra (negative mode) of resveratrol monolaurate (before and after purification) (**A**): Crude sample of resveratrol monolaurate; (**B**) Purified sample of resveratrol monolaurate.

The negative mode of ESI source of MS was employed to confirm the synthesis of monoesters. Di- and triesters were identified by comparing their elution time to monoesters by HPLC (data not given here) and NMR because of poor ionization of triesters, especially the triesters with long-chain acyl tail, under the current ESI conditions. In Figure 1 and Figure S1 (Supplementary Materials), MS results of resveratrol monolaurate samples before and after purification are also provided as an example, which shows the MW (molecular weight) of resveratrol and its monolaurate at 228 and 410 Da.

In addition to the HPLC and MS analyses, it was necessary to investigate the esterification location on the hydroxyl groups of resveratrol molecules. For a given enzyme, enantioselectivity, regioselectivity and acylation rate are usually affected by other reaction conditions as well as the structures of acyl acceptors and the acyl donors [30]. However, when the acyl donors are the only variable that shares the same trend of the fundamental structure and functional moiety, such as various aliphatic acids with different chain lengths, it may pose no significant effect on the regioselectivity of lipase-catalysed synthesis of the same type of esters (e.g., monoesters) [31,32]. The current study was not focused on the effect of fatty acyl chain length on the regioselectivity of acylation of resveratrol. Resveratrol monocaprylate, one of the medium-chain fatty acid esters of resveratrol, was selected for further structure elucidation. The purification of resveratrol esters, including its monocaprylate, was conducted as previously reported with slight modification [23]. Crude products of resveratrol monocaprylate were separated by flash column chromatography using silica gel as the stationary phase, and hexane, ethyl acetate, and formic acid by gradient as the mobile phase, and each fraction was monitored via TLC (hexane/ethyl acetate/formic acid at 3:3:0.1, *v*/*v*/*v*).

The formula of acylated resveratrol was confirmed by using different types of NMR (^1^H NMR, HSQC, COSY) after being separated by flash column chromatography. The acylation position of resveratrol caprylate was determined by comparison with the literature data on resveratrol and resveratrol monoacetate, diacetate, and triacetate [17,33]. For original resveratrol, H-2/6, H-3′/5′, and H-2′/6′ are the symmetric hydrogens and gave chemical shifts at 6.40, 6.77, and 7.40, respectively. Although monoester samples were all a mixture of these two types of isomers, since their actual separation from each other using the current purification technique (silica gel chromatography) is fairly difficult, a high-resolution NMR (Bruker Avance 500 Hz) conducted with lower temperature and the addition of 1.2% (mole percentage of the sample) picric acid can help to narrow the hydrogen peaks of phenolic hydroxyl groups and determine these two monoester isomers in a mixture sample [34]. As is known, acylation on 4′-OH not only removes the chemical shift of ^1^H NMR on position 4′ but also decreases the electron-donating effect of 4′-OH, which, consequentially, significantly increases the chemical shifts of hydrogens around the C-4′ position, especially 3′/5′-H. Similarly, acylation of 3-OH gives a larger chemical shift for hydrogens around the C-3 position, especially 2/4-H. Meanwhile, 3-OH acylation makes acylated resveratrol an asymmetric molecule—it differentiates the chemical shifts of 2-H and 6-H instead of the unified one in the 4′-monoester (Table 2 and Figure S3). For the triester of resveratrol, the chemical shifts of all the hydrogens moved to the low field (increased value), and the hydrogens next to acylation points moved the most. Furthermore, COSY and HSQC modes were also employed to identify the acylation point of resveratrol derivatives, but not much extra information was obtained. The diesters of resveratrol could be easily confirmed as 3,4′- and 3,5-diesters according to the monoacylation positions. Chemical acylation has been considered less or not regioselective compared to enzymatic acylation, according to previous studies [30,35,36,37].

### 2.3. Evaluation of Antioxidant Efficacy of Resveratrol Esters

Oxidation is one of the significant causes of food deterioration and nutrition/quality loss during manufacturing and storage, especially of processed food rich in unsaturated lipid and proteins/peptides. Lipid oxidation occurs due to the unsaturation of lipids that yields lipid peroxides and generates various volatile aldehydes/ketones/alcohols/acids that lead to the development of an off flavour [38]. Oxidative damage of peptides/proteins takes place at their side-chain residue. It induces thiol oxidation, aromatic hydroxylation, and formation of carbonyl groups, which results in the conformational changes (fragmentation, aggregation, and polymerization) of both the secondary/tertiary structures of the protein and is related to the eating quality traits of certain foods [39]. These oxidation processes decrease the nutritional quality of food and expose consumers to a series of safety/health risks (e.g., oxidative stress and inflammation), which eventually impairs consumers’ purchasing intention. Therefore, various processing and preservation strategies have been developed to prevent the oxidative deterioration of protein and especially lipid-rich food products. These include physical methods such as altering packaging atmosphere, encapsulating, and emulsifying, and chemical processes such as adjusting pH and using antioxidants. The addition of food antioxidants is often both economical and feasible. However, the desire of replacing synthetic antioxidants with naturally derived substances, especially dietary polyphenols, has been of interest for decades due to their safety record and compatibility with the human body, although synthetic antioxidants have remained in the current commercial market due to their advantage of price and efficacy. Thus, the list of alternatives to synthetic lipidic antioxidants has remained relatively unchanged. Ascorbyl palmitate, mixed tocopherols (mixture of artificial and natural α/β/γ-tocopherol), deodorized rosemary extract, and green tea extract are the main naturally derived alternatives for the pure artificial antioxidants such as BHA (butylated hydroxyanisole), BHT (butylated hydroxytoluene), and TBHQ (tert-butylhydroquinone). For the manufacturers of lipid-based food, supplements, and nutraceuticals, the application of certain natural phenolics is usually compromised due to their limited lipophilicity.

Acylation of natural phenolics such as resveratrol can effectively enhance lipophilicity and extend their application in lipidic media (Table 3). Meanwhile, when the total molecular weight (MW) of compounds is under a certain range (<500 Da), acylation can enhance oral bioavailability via reducing the number of exposed hydroxyl groups that belong to hydrogen bond donors (‘Lipinski’s rule’ and ‘rule of three’) [40]. Such a deduction has been verified via pharmacokinetic studies of certain dietary phenolics [41,42,43]. Although acylation of polyphenolics exhibits tremendous benefits, loss of exposed hydroxyl groups may also influence their original antioxidant ability. To the best of our knowledge, this is the first contribution incorporating resveratrol monoesters, diesters, triesters with varying acyl chain lengths (C2:0-C18:0) into an in vitro antioxidant analysis. In the following discussion, antioxidant efficacies of mono-/di-/tri-esters of resveratrol based on four in vitro chemical-based assays, DPPH, ABTS, FRAP, and ferrous chelation, are evaluated for further comparison and analysis.

#### 2.3.1. DPPH-Scavenging Assay

Free radicals, including ROS and RNS, can be defined as any molecule or species that exist independently with an unpaired electron; they are highly reactive and can damage/modify in vivo functional biomolecules or in vitro nutritional components, causing deterioration of human health and food quality. DPPH is a classic nitrogen radical that is highly accessible and characterized by stable deep-violet colour when dissolved in alcohol. It has hydrogen-acceptor capability to antioxidants and is used in DPPH assays to determine the radical scavenging ability of various biological substrates. During radical scavenging, the violet colour of the solution is faded into pale yellow, and DPPH radicals are neutralized. The efficacy of radical scavenging can be measured using the UV mode of microplate spectrophotometers at 517 nm or by using electron paramagnetic resonance (EPR) spectroscopy. The DPPH-scavenging ability is expressed as mole efficacy equivalents of Trolox per mole of the sample (%, *n*/*n*).

**Figure 2:**
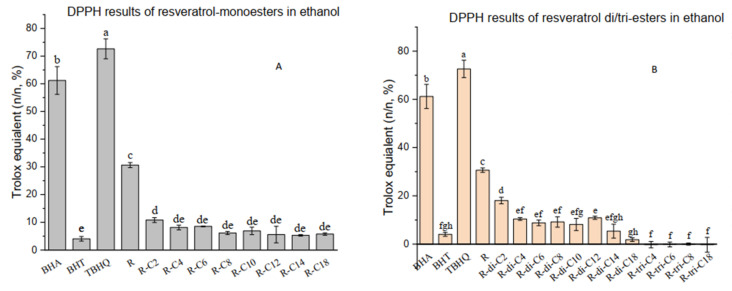
DPPH radical-scavenging capacity. (**A**): DPPH radical scavenging capacity in mole efficacy equivalent of Trolox per mole of resveratrol (R) or its monoesters (%, *n/n*). R-C2, R-C4, R-C6, R-C8, R-C10, R-C12, R-C14, R-C18 are resveratrol esters of acetic, butyric, caproic, caprylic, capric, lauric, myristic, and stearic acids, respectively. (**B**): DPPH radical scavenging capacity in mole efficacy equivalent of Trolox per mole of resveratrol (R) or its di-/triesters (%, *n/n*). R-di-C2, R-di-C4, R-di-C6, R-di-C8, R-di-C10, R-di-C12, R-di-C14, and R-di-C18 are resveratrol diesters of acetic, butyric, caproic, caprylic, capric, lauric, myristic, and stearic acids, respectively; R-tri-C4, R-tri-C6, R-tri-C8, and R-tri-C18 are resveratrol triesters of caproic, caprylic, capric, and stearic acids, respectively. Each assay was replicated 3 times. Entries with different letters were significantly different at *p* < 0.05, performed by Tukey’s HSD test.

For resveratrol monoesters (Figure 2A), their DPPH scavenging efficacy per mole ranges from 5.28 to 10.81% of the scavenging efficacy of Trolox. These values are around or less than one-third of the resveratrol (30.62%). Meanwhile, resveratrol monoacetate exhibits a slightly but not significantly higher value compared to the rest of monoester derivatives. As shown in Figure 2B, the resveratrol triesters exhibit no scavenging efficacy, while the diester derivatives of resveratrol generally show better antioxidant performance (1.86–18.05%). However, similar to other radical scavenging tests, a significant disadvantage of the DPPH-scavenging assay is that this discoloration assay takes time (30 min to hours) to reach an endpoint, and the measurements at different time points can immensely vary for the same sample. Therefore, the interpretation of the results sometimes makes more sense in evaluating the scavenging rate with the same reaction time instead of the final scavenging percentage. However, it still provides a mean to rank the reaction rate of different antioxidants. Herein, the result implies that the quantity of the exposed hydroxyl group of resveratrol derivatives does not dominate the DPPH radical-scavenging rate. Instead, a combination effect of solvent polarity/partition coefficient and the molecular affinity between DPPH radicals and resveratrol derivatives may play a more critical role. Besides, long acyl chains may hinder the neutralization reaction between radicals and acylated resveratrol molecules, while short-chain acyl tails do not suffer from this problem (e.g., R-C2 and R-di-C2). The trends in DPPH results of the monoesters are not entirely in line with those of Oh et al. [23], in which the EPR method was used rather than UV spectrum, and the optimal length (C18:0 and C18:1) of acyl chain of resveratrol derivatives can be found with the highest antioxidant efficacy. Furthermore, the overall values of the DPPH-scavenging assay of resveratrol and its products are far lower than those of BHA and TBHQ.

#### 2.3.2. ABTS^+^-Scavenging Assay

The ABTS assay is considered one of the most sensitive techniques to identify antioxidant activity because its neutralization by antioxidants involves faster reaction kinetics [44]. Similar to the DPPH assay, both HAT (hydrogen atom transfer) and SET (single electron transfer) mechanisms apply to the neutralization of ABTS^+^, but the difference is that log P of DPPH is 4.35 while that of ABTS is more hydrophilic, at about 0.43 [23]. This assay was initially developed by Miller et al. [45] to monitor the antioxidant status of bodily fluids in premature neonates. ABTS yields a bluish-green radical cation, ABTS^+^, once being oxidized by K_2_S_2_O_8_ in a highly polar solvent. This radical dissolves in both water and alcohol, therefore it is a suitable method for investigating the effect of polarity of a solvent on the antioxidant evaluation of resveratrol derivatives. In this test, the radical-scavenging results are visualized by discoloration reaction occurs when antioxidants reduce the ABTS radical cation.

**Figure 3:**
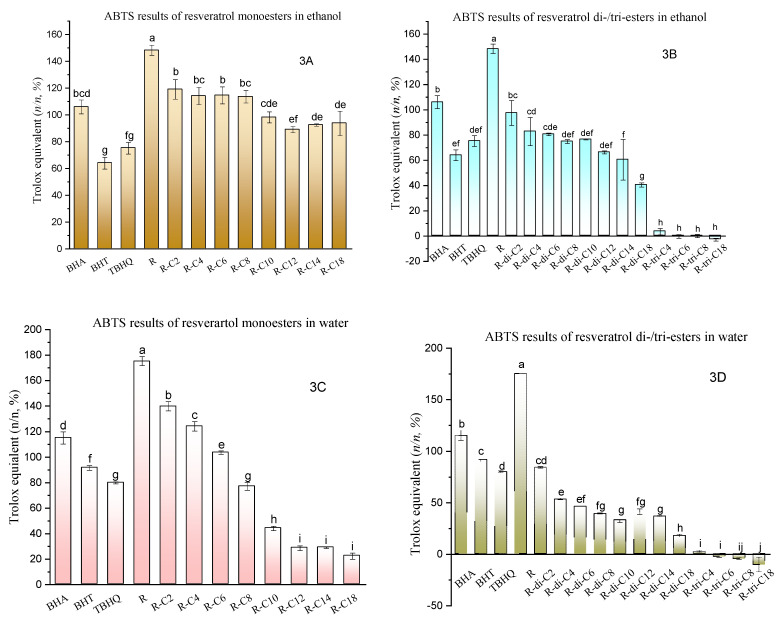
ABTS cation radical-scavenging capacity (**A**): ABTS radical scavenging capacity in mole efficacy equivalent of Trolox per mole of resveratrol (R) or its monoesters (%, *n/n*) in ethanol system. R-C2, R-C4, R-C6, R-C8, R-C10, R-C12, R-C14, R-C18 are resveratrol esters of acetic acid, butyric, caproic, caprylic, capric, lauric, myristic, and stearic acids, respectively. (**B**): ABTS radical scavenging capacity in mole efficacy equivalent of Trolox per mole of resveratrol (R) or its di-/triesters (%, *n/n*) in ethanol system. R-di-C2, R-di-C4, R-di-C6, R-di-C8, R-di-C10, R-di-C12, R-di-C14, and R-di-C18 are resveratrol diesters of acetic acid, butyric, caproic, caprylic, capric, lauric, myristic, and stearic acids, respectively; R-tri-C4, R-tri-C6, R-tri-C8, and R-tri-C18 are resveratrol triesters of caproic, caprylic, capric, and stearic acids, respectively. (**C**): ABTS radical scavenging capacity in mole efficacy equivalent of Trolox per mole of resveratrol (R) or its monoesters (%, n/n) in water system. R-C2, R-C4, R-C6, R-C8, R-C10, R-C12, R-C14, R-C18 are resveratrol esters of acetic, butyric, caproic, caprylic, capric, lauric, myristic, and stearic acids, respectively. (**D**): ABTS radical scavenging capacity in mole efficacy equivalent of Trolox per mole of resveratrol (R) or its di-/triesters (%, n/n) in water system. R-di-C2, R-di-C4, R-di-C6, R-di-C8, R-di-C10, R-di-C12, R-di-C14, and R-di-C18 are resveratrol diesters of acetic, butyric, caproic, caprylic, capric, lauric, myristic, and stearic acids, respectively; R-tri-C4, R-tri-C6, R-tri-C8, and R-tri-C18 are resveratrol triesters of caproic, caprylic, capric, and stearic acids, respectively. Each bar was replicated 3 times. Bars with different letters were significantly different at *p* < 0.05, performed by Tukey’s HSD test.

Similarly, ABTS radical cation-scavenging ability is expressed as mole efficacy equivalents of Trolox per mole of resveratrol or its esters (%, *n*/*n*). When assays were conducted in ethanol, the ethanol-phase ABTS^+^-scavenging results of monoesters of resveratrol ranged from 92.52 to 119.08% of the scavenging efficacy of Trolox, which are comparable to that of Trolox but lower than that of the original resveratrol molecule (Figure 3A). Compared to the results of monoesters in ethanol, the scavenging efficacy of diesters is generally lower and ranges from 40.41 to 97.41% (Figure 3B). Meanwhile, resveratrol derivatives substituted with a shorter acyl chain show a higher scavenging effect (Figure 3A–D).

Unlike the DPPH results, ethanol-phase ABTS tests of resveratrol monoesters and diesters display similar or higher radical-scavenging efficacy than BHA, BHT, and TBHQ (Figure 3A,B). However, when the water-phase ABTS assays were used to measure the same samples, the activity of monoesters with medium-long-chain acyl groups became much lower than those of BHA, BHT, and TBHQ (Figure 3C). Compared with the ethanol-phase ABTS test, the efficacy of R-C10, R-C12, R-C14, R-C18 in water-phase trials fell to 44.07, 28.62, 28.96, and 22.29%, respectively, while efficacy of R-C2 and R-C4 increased to 139.84 and 124.18%, respectively (Figure 3C). Similar trends modulated by the acyl-chain length can also be found in the test result of diester derivatives (Figure 3D). Moreover, water-phase ABTS tests enlarged the difference in antioxidant efficacy of monoesters with different acyl-chain lengths that exist in the ethanol-phase result of monoesters (Figure 3A,C). This efficacy difference of monoesters was narrowed down when assays were conducted in a more lipophilic solvent (ethanol) that better dissolved and dispersed the monoesters. When the solvent system (water) imposed lower solubility on the samples (diesters), their antioxidant efficacy was lowered compared to the same sample (diesters) tested in a high-solubility solvent (ethanol), probably because less contact is possible between antioxidants and radicals (Figure 3B,D). Therefore, the solubility of tested antioxidants in the solvent system significantly affects antioxidant efficacy in the ABTS tests. Overall, the antioxidant activity of resveratrol esters was found to be negatively associated with acyl chain length and degree of acylation in both solvent systems. The higher the degree of acylation or the longer the acyl chain length of esters, the lower the efficacy was. This may be explained by an increased lipophilicity, and/or that increased steric hindrance of esters retards radical-scavenging efficacy.

As expected, the triesters showed no efficacy in both DPPH- and ABTS^+^-scavenging tests (Figure 3B,D). However, this is not to say that the triester derivatives of resveratrol cannot be used as antioxidant supplement, cosmetic, or pharmaceuticals ingredients. The esterified phenolics may be hydrolysed by the microorganism, biased pH, and enteric/in vivo enzymes under specific physiochemical or physiological environments [41,46]. Depending on the acyl chain length and degree of acylation, they may be rapidly hydrolysed, detoxified, and excreted from the human body before/after entering systemic circulation and act as antioxidants in this process [47,48]. Alternatively, they may be more tolerable to the degradation, hydrolysis and/or detoxification so that they can stay in the target sites such as skin surface and circulatory system longer and be slowly deacylated to function as a slow-release radical scavenger. Nevertheless, resveratrol triesters may not be recommended for the antioxidant preservation of edible oils or other water/alcohol-free matrices, attributed to the fact that fully acylated resveratrol cannot readily be deacylated in such an environment to exert its antioxidant capability.

#### 2.3.3. Ferric-Reducing Antioxidant Power, FRAP

The FRAP was initially developed to measure the redox status of plasma, consequently giving it the full name of the ferric reducing ability of plasma [49]. The assay detects compounds with reducing potentials less than 0.7 V (the reducing potential of Fe^3+^-TPTZ complex), which is quite close to that of ABTS^+^ (0.68 V) [50]. However, the FRAP purely functions with SET rather than HAT mechanism. It cannot be used for testing the antioxidants quenching radicals via only HAT, such as glutathione (-thiol). This factor contributes to underestimating the samples rich in thiols, including various body fluids (e.g., intracellular, interstitial, and intravascular fluids). Besides, the FRAP test is conducted under acidic conditions (pH 3.6), while most antioxidant assays are in a neutral system. Therefore, FRAP values usually show a poor correlation with other antioxidant results [51]. Furthermore, due to the mechanism difference, the FRAP test is a method for distinguishing the main antioxidant mechanisms of the compounds when used in combination with other antioxidant tests.

**Figure 4:**
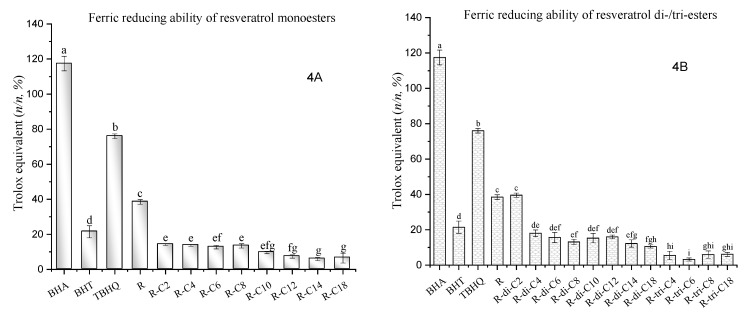
Ferric-reducing antioxidant power of resveratrol esters. (**A**): Ferric reducing antioxidant power in mole efficacy equivalent of Trolox per mole of resveratrol (R) or its monoesters (%, *n/n*). R-C2, R-C4, R-C6, R-C8, R-C10, R-C12, R-C14, R-C18 are resveratrol esters of acetic, butyric, caproic, caprylic, capric, lauric, myristic, and stearic acids, respectively. (**B**): Ferric reducing antioxidant power in mole efficacy equivalent of Trolox per mole of resveratrol (R) or its di-/triesters (%, *n/n*). R-di-C2, R-di-C4, R-di-C6, R-di-C8, R-di-C10, R-di-C12, R-di-C14, and R-di-C18 are resveratrol diesters of acetic, butyric, caproic, caprylic, capric, lauric, myristic, and stearic acids, respectively; R-tri-C4, R-tri-C6, R-tri-C8, and R-tri-C18 are resveratrol triesters of caproic, caprylic, capric, and stearic acids, respectively. Each assay was replicated 3 times. Entries with different letters were significantly different at *p* < 0.05, performed by Tukey’s HSD test.

Figure 4A shows that FRAP values of monoester derivatives range from 5.92 to 14.12% of the reducing efficacy of Trolox. Resveratrol monoesters with short- and medium-chain acyl tails (R-C2, R-C4, R-C6, R-C8) gave significantly higher values than monoesters with medium- and long-chain tails (R-C12, R-C14, R-C18). Meanwhile, monoesters show a considerably lower ferric-reducing power than resveratrol and purely synthetic antioxidants, especially BHA and TBHQ. A similar trend can be noticed in the results of diesters (Figure 4B). The FRAP values of diesters range from 5.50 to 39.58%. The reducing power of diesters is generally lower than that of resveratrol, BHA, and BHT. However, resveratrol diacetate (R-di-C2) is an exception that exhibits an almost equal power to resveratrol. Furthermore, long-chain diesters (R-di-C18) showed a significantly lower reducing efficacy than short-chain diesters (R-di-C2 and R-di-C4). Similar to the result of ABTS assays, lengths of acyl chain and degree of acylation, namely lipophilicity and steric hindrance/steric accessibility, play essential roles in reducing ferric ions. Additionally, the trends and relative mole efficacy equivalents of Trolox of BHA, BHT, TBHQ, resveratrol, and related derivatives between the DPPH and FRAP assays were pretty close. This may be explained by the similarity of the action mechanism between these two assays. Even though the DPPH test utilizes both SET and HAT mechanisms, HAT is suggested to be relatively marginalized [50].

For triesters, they surprisingly exhibit remarkable reducing power, which implies significant hydrolysis happens to the triesters in the aqueous acidic solution. However, this hydrolysis reaction may also occur for monoesters and diesters. Thus, this fact needs to be considered when interpreting the rest of the FRAP results. Worthy of note, this FRAP test is rarely used for evaluating the antioxidant efficacy of resveratrol fatty acid esters. Thus, we notice that it may not serve for testing hydrolysable compounds such as various acylated derivatives if such hydrolyses are not preferred or expected in targeted applications.

#### 2.3.4. Ferrous Ion-Chelation Ability

Phenolic antioxidants are usually capable of turning ferric ions into ferrous ions. However, ferrous ion is a key transition metal ion responsible for the initiation of peroxidation in food and biological systems. It contributes to the generation of hydroxyl radicals via the Fenton reaction. This leads to the deterioration of nutritional/functional components or biomolecules in food/cosmetic/nutraceutical products or human health. Certain phenolic compounds can be considered adequate secondary antioxidants if they can lower the concentration of free metal ions available for catalysing peroxidation [52]. In this assay, ferrozine and free ferrous ion can form a purple Fe^2+^-ferrozine complex with max absorbance at 562 nm, and the colour intensity of the complex decreases with the increase in concentration of chelating agents.

**Figure 5:**
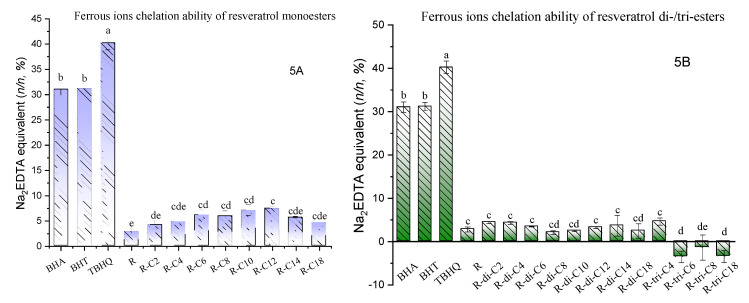
Ferrous ion-chelation ability of resveratrol esters. (**A**): Ferrous ions chelation ability in mole efficacy equivalent of EDTA per mole of resveratrol (R) or its monoesters (%, *n/n*). R-C2, R-C4, R-C6, R-C8, R-C10, R-C12, R-C14, R-C18 are resveratrol esters of acetic, butyric, caproic, caprylic, capric, lauric, myristic, and stearic acids, respectively. (**B**): Ferrous ions chelation ability in mole efficacy equivalent of EDTA per mole of resveratrol (R) or its di-/triesters (%, *n/n*). R-di-C2, R-di-C4, R-di-C6, R-di-C8, R-di-C10, R-di-C12, R-di-C14, and R-di-C18 are resveratrol diesters of acetic, butyric, caproic, caprylic, capric, lauric, myristic, and stearic acids, respectively; R-tri-C4, R-tri-C6, R-tri-C8, and R-tri-C18 are resveratrol triesters of caproic, caprylic, capric, and stearic acids, respectively. Each experiment was replicated 3 times. Entries with different letters were significantly different at *p* < 0.05, performed by Tukey’s HSD test.

Figure 5A,B shows that the Fe^2+^-chelation abilities of resveratrol and its derivatives are relatively lower than those of Trolox and pure artificial antioxidants. The monoesters exhibit chelation efficacy ranging from 4.14 to 7.41% of the chelation efficacy of EDTA, while for diesters, they range from 2.08 to 4.43%. Very different to the DPPH, ABTS, and FRAP results, monoacylation overall does not negatively affect the chelation ability of resveratrol (2.81 ± 0.58%). Contrarily, monoesters with a medium-length acyl chain (R-C6, R-C8, R-C10, and R-C12) display a significantly higher chelation efficacy than nonacylated resveratrol (Figure 5A). A similar trend applies to diacylation, where there is no significant difference among values of diesters or those between diesters and resveratrol. Meanwhile, another special case is that resveratrol tricaproate, tricaprylate, and tristearate all exhibit negative values of chelation ability, which is most probably because of the insolubility and precipitation of these triesters instead of an appropriate dispersion in the system, which leads to a higher absorbance at 562 nm. However, this phenomenon did not happen to the less lipophilic resveratrol tributyrate (R-tri-C4). Along with the significantly higher values of R-C6/C8/C10/C12, these highly varied antioxidant results that are affected by the length of the acyl chain of phenolic esters have been reported in previous studies [38]. There are three prominent cases: (1) in some instances, the antioxidant activity increases with the increase in the alkyl/acyl chain length to a maximum point (the cutoff point) and then starts to reverse with further chain length extension [23]; (2) the antioxidant activity may also consistently decrease with the increase in chain length if this maximum point starts with the phenolic esters with the shortest acyl chain (formyl) [53]; or (3) the antioxidant activity may consistently increase with the increase in chain length if the cutoff point ends with/after phenolic esters with the longest acyl chain (stearyl) [54]. In this study, although case 1 was found in the trends of Figure 5A, it is more likely a situation of case 2 (Figure 2B, Figure 3A–D, Figure 4A,B). In brief, the cutoff phenomenon may be well-matched with the ‘polar paradox’ theory [24]. An optimal antioxidant mainly resulted from the solubility/partition coefficient of solvent and reactants, the steric hindrance and other structural properties of reactants, and the amphiphilicity of substrates, which are all biased by the length of the acyl chain.

## 3. Materials and Methods

### 3.1. Materials

Resveratrol was obtained from Xi’an Yuensun Biological Technology Company Limited (Xi’an, Shaanxi, China) and eight vinyl fatty acids (stabilized with MEHQ, 99.0+%) including vinyl acetate, vinyl butyrate, vinyl caproate, vinyl caprylate, vinyl caprate, vinyl laurate, vinyl myristate, and vinyl stearate, were purchased from TCI America^TM^. Lipase PS was obtained from Amano Pharmaceutical Co., Ltd. (Tokyo, Japan). Silica gel and flexible thin-layer chromatography (TLC) plates with silica gel 60A (2.5 × 7.5 cm, layer thickness of 250 μm) were bought from Selecto Scientific (Suwanee, GA, USA). Perdeuterated dimethyl sulfoxide (DMSO-d6) containing tetramethylsilane (TMS) was purchased from Cambridge Isotope Laboratories, Inc. (Andover, MA, USA). DPPH (2,2-diphenyl-1-picrylhydrazyl), ABTS [2,2′-azino-bis(3-ethylbenzothiazoline-6-sulfonic acid)], TPTZ (1,3,5-tri(2-pyridyl)-2,4,6-triazine), ethylenediaminetetraacetic acid trisodium salt (Na_3_EDTA), potassium persulfate (K_2_S_2_O_8_), ferrozine (3-(2-pyridyl)-5,6-diphenyl-1,2,4-triazine-4,4-disulfonic acid sodium salt), ferric chloride (FeCl_3_), ferrous chloride (FeCl_2_), Trolox, and picric acid were obtained from Sigma-Aldrich Canada, Ltd. (Oakville, ON, Canada). Organic solvents and other chemicals, including analytical grade DMF (dimethylformamide), ethyl acetate, hexane, methanol, acetonitrile, water, formic acid, and NaOH (sodium hydroxide) were from Merck (Darmstadt, Germany), Sigma-Aldrich Canada Ltd. (Oakville, ON, Canada) or Fisher Scientific Ltd. (Ottawa, ON, Canada) and were used without any further purification. All solvents used were of ACS or MS grade.

### 3.2. Enzymatic Preparation of Resveratrol Monoesters and Chemical Preparation of Resveratrol Di-/Tri-esters

Resveratrol (30 mM, 691–709 mg) and vinyl fatty acids (100 mM, ~869–3220 mg) were incubated in DMF (100 mL) in 200 mL sealed conical flasks under a blanket of nitrogen at 50 °C in an orbital-shaking water bath (200× *g*) in the dark. For enzymatic synthesis, the enzyme was added to a final concentration of 5 mg/mL, and for alkaline synthesis, sodium hydroxide was added to a final concentration of 0.2%. The reaction progress was monitored by using TLC plates and ESI-ToF-MS (electrospray ionization-time-of-flight-mass spectrometry). After 12 h enzymatic or 1–2 h alkaline reaction, the mixture was cooled, filtered, and dried for purification and NMR (nuclear magnetic resonance) analysis. The details and parameters of MS/NMR analysis and purification process are given below.

### 3.3. HPLC and MS Analysis of Resveratrol Derivatives

The chemical structures of resveratrol monoesters were confirmed by MS, and those of resveratrol diesters/triesters via TLC, HPLC, and/or NMR.

HPLC conditions: Agilent 1100 HPLC system consisted of a degasser, a binary pump Bin Pump SL, a thermostatted HiP-ALS autosampler, a TCC SL column oven, and a DAD detector (Agilent Technologies, Palo Alto, CA, USA). Separation was carried out using an Agilent Eclipse XDB-C18 column (4.6 mm × 250 mm × 5 μm). The mobile phase consisted of 0.1% formic acid in deionized water (A) and 0.1% formic acid, and 5% acetonitrile in methanol (B). The solvent gradients were varied according to the compounds’ acyl chain length (see the Results and Discussion). The column was thermostatically controlled at 40 °C, and the flow rate was set at 1 mL/min. The UV-visible absorbance of the peaks was monitored between 190 and 400 nm. All samples and standards were diluted in acetonitrile, and the sample injection volume was 5 μL.

MS conditions: Agilent ToF–MS detector system (Agilent Technologies, Palo Alto, CA, USA) with electrospray ionization (ESI) in a negative ion mode and full-scan mass spectral data was used over an *m*/*z* range of 100 to 1700 for identification of each fraction. The MS conditions were as follows: drying gas flow rate, 5 L/min; nebulizer pressure, 60 psi; drying gas temperature, 350 °C; and capillary voltage, +3.5 kV. The MS data were acquired and analysed by the MassHunter Acquisition B.03.01, Qualitative Analysis B.03.01. Only the ions with a charge-to-mass ratio (*m*/*z*) showing systematic differences less than 10 ppm between detection and calculation values were preliminarily considered as the potential target products (resveratrol esters).

### 3.4. Purification and NMR Analysis of Resveratrol Derivatives

Resveratrol derivatives were purified according to a previous protocol [23] using silica gel column chromatography. The solvent used contained gradients of hexane/ethyl acetate/formic acid (90:10:2, 80:20:2, 70:30:2, and 60:40:2, *v*/*v*/*v*). Each fraction was collected and monitored by silica gel-coated TLC (hexane/ethyl acetate/formic acid, 3:3:0.12, *v*/*v*/*v*). Each purified compound was procured following solvent removal via evaporation under a stream of nitrogen or using vacuum rotary evaporation.

Proton nuclear magnetic resonance (^1^H NMR), correlation spectroscopy (COSY), and heteronuclear single-quantum correlation (HSQC) spectroscopy were used to identify their molecular structures and esterification position. All NMR data were collected on a Bruker Avance 300 MHz or/and 500 MHz (Bruker Biospin Co., Billerica, MA, USA), and data interpretation was performed with Topspin 3.0 with ICON and MestReNova (Mestrelab Research SL, Santiago De Compostela, Spain). The samples were dissolved at a concentration of 25 mg/mL in DMSO-d6 containing TMS as internal standard. The results were confirmed by comparing the chemical shifts of resveratrol and its derivatives.

### 3.5. Antioxidant Activity

#### 3.5.1. DPPH Radical-Scavenging Activity

The DPPH assay was conducted by using a modified method reported earlier [51]. Briefly, 200 μL of DPPH methanolic solution (350 μM) was mixed with 10 μL of test samples or a series of Trolox standards (12.5–1200 μM) in a 96-well plate and allowed to react at room temperature for 30 min in the dark before the absorbance was recorded at 515 nm. All samples were prepared as ethanolic solutions at a concentration of 100 μM and tested in triplicate. DPPH antioxidant activities were calculated as mole efficacy equivalents of Trolox per mole of resveratrol esters (*n*/*n*, %).

#### 3.5.2. ABTS Radical-Scavenging Activity

The ABTS radical scavenging activity was evaluated as previously reported [51] with slight modification. Stock solution included 0.2 mL ABTS solution (7.4 mM), 0.2 mL oxidant solution (potassium persulfate, 2.6 mM) and 0.1 mL Trolox solution (10 mM). ABTS^+^ working solution was prepared by reacting ABTS stock solution with oxidant solution in a ratio of 1:1 (*v*/*v*) in the dark for 12 h before use. The ABTS^+^ working solution was diluted with ethanol or water to the absorbance of 0.70 ± 0.05 at 406 nm. Five microliters of samples (100 μM) or Trolox standard (12.5–1200 μM) were reacted with 200 μL of ABTS^+^ working solution in the dark at room temperature for 6 min in a 96-well plate, and the absorbance was read at 734 nm. All measurements were conducted in triplicate. ABTS radical-scavenging activities were expressed as mole efficacy equivalents of Trolox per mole of resveratrol esters (*n*/*n*, %).

#### 3.5.3. Ferric-Reducing Antioxidant Power (FRAP) Assay

The FRAP assay was performed as reported previously [51,55]. Five microliters of samples (100 μM) or standards were reacted with 180 μL of ferric-TPTZ reagent (prepared by mixing 300 mM acetate buffer, pH 3.6, 10 mM TPTZ in 40 mM HCl with 20 mM FeCl_3_·6H_2_O at a ratio of 10:1:1 (*v*/*v*/*v*)) which was warmed to 37 °C before use. The absorbance of the reaction mixture was then read at 595 nm. The standard curve was prepared using Trolox ranging from 15.625 to 1000 μM. All measurements were assessed in triplicate. Results were expressed as mole efficacy equivalents of Trolox per mole of resveratrol esters (*n*/*n*, %).

#### 3.5.4. Ferric Ion-Chelation Ability

The ability of resveratrol esters to chelate ferrous Ions was measured according to Ambigaipalan et al. [56] with certain modifications. Ten microliters of samples (100 μM) or Na_3_EDTA standards (12.5–2000 μM) were first mixed with 20 μL FeCl_2_ (1 mM), which was then allowed to mix with 200 μL ferrozine (0.5 mM) in a 96-well plate. The mixtures were subsequently kept at room temperature with thorough shaking for 10 min. The absorbance of the resulting solution was read at 562 nm. The ferrous ion-chelation ability of samples was calculated as mole efficacy equivalents of EDTA per mole of resveratrol esters (*n*/*n*, %).

### 3.6. Statistic Analysis

All determinations were replicated three times and expressed as mean values with standard deviations. One-way ANOVA followed by Tukey’s HSD test using SPSS 24.0 for Windows (SPSS Inc., Chicago, IL, USA) was used to determine statistical differences at *p* < 0.05.

## 4. Conclusion

Enzymatic acylation of resveratrol generated monoesters as the main products, and base-catalysed acylation afforded diesters and triesters. Nuclear magnetic resonance (NMR) spectroscopy identified resveratrol tricaprylates and two isomers of resveratrol monocaprylates, and mass spectrometry (MS) was used to determine the resveratrol monolaurate. Reveratrol was successfully lipophilized via transesterification, as calculated using the online lipophilicity calculator ‘ALOGPS 2.1′. In DPPH, ABTS, and FRAP assays, resveratrol exhibited a higher antioxidant efficacy than its acylated analogues, while in the ferrous chelation test, acylated resveratrols demonstrated a comparable or even better chelation efficacy than resveratrol. Among resveratrol monoesters, compounds with short- or medium-chain acyl groups, especially monoacetate ester, showed the highest scavenging activity in DPPH and ABTS assays. For resveratrol diesters, the acetate ester showed the highest efficacy in DPPH, ABTS, and FRAP tests. However, in ferrous chelation assays, the acyl groups for obtaining the optimal effectiveness as a monoester were caproate, caprylate, caprate, and laurate. Generally, triesters show no significant chemical in vitro antioxidant activity, unless when a low pH level is used as the solvent system of FRAP. However, the resveratrol tributyrate in the ferrous chelation assay is an exception. The antioxidant efficacy of monoesters is overall better than that of diesters and triesters, although the result of resveratrol diacetate in the FRAP test is exceptional. This study suggests that the lengths of the acyl chain and the degree of acylation of resveratrol esters significantly influence their antioxidant activity trends in radical-scavenging, ferric ion-reducing, and ferrous ion-chelation experiments. The steric accessibility and partition coefficient of antioxidants as well as the pH and solubility of the solvent system of assays have to be considered during interpretation of their antioxidant performance.

## Figures and Tables

**Table 1:** General reaction conditions and yields of enzymatically synthesized resveratrol esters.

Resveratrol	Acyl Donors	Products (yield)	Enzyme	Solvent	Other Reaction Conditions	Reference
500 mg, 182.75 mM	vinyl acetate (2 mL, 181.01 mM)	resveratrol 4′-O-acetate (40%)	Novezym 435, 500 mg	tert-amyl alcohol (10 mL)	40 °C, 400 rpm, 90 h	[27]
0.5 mg, 14.62 mM	vinyl acetate (50 μL, 3.62 M)	resveratrol 4′-O-acetate (50%)	Novezym 435, 4–6 mg	2-methyl-2-butanol (100 μL)	37 °C, 250 rpm, 72 h	[28]
	vinyl caprate (50 μL, 1.48 M)	resveratrol 4′-O-cprate (10%)			37 °C, 250 rpm, 24 h	
	vinyl cinnamoate (50 μL, 2.11 M)	resveratrol 4′-O-cinnamoate (trace)			37 °C, 250 rpm, 240 h	
30 mg, 32.89 mM	vinyl acetate (25% volume of solution, 1 mL, 2.72 M)	resveratrol 4′-O-acetate (45%)	Novezym 435, 4–5 times of total substrate	2-methylbutan-2-ol (3 mL)	37 °C, 250 rpm, 72 h	
22.8 mg, 50 mM	vinyl acetate (508.75 μL, 189.41 M)	resveratrol 4′-O-acetate (95.2%)	Novezym 435 (549.2 mg)	2-methyl-2-butanol (2 mL)	60 ℃, 40 kHz ultrasonication, 147.8 W, 10.78 h	[29]
22.8 mg, 0.50 mM	vinyl acetate (92.5 μL, 4.30 M)	resveratrol 4′-O-acetate (83.5%)	Lipase CSL (100.0 mg)	MTBE (20 mL)	35 °C, 400 W, microwave irradiation, 4 h	[25]
		3-OH > 4′-OH, resveratrol monoacetate (54.9%)	Lipase PS (100.0 mg)			
		3-OH ≈ 4′-OH, resveratrol monoacetate (27.5%)	Lipase AKL (100.0 mg)			
		3-OH < 4′-OH, resveratrol monoacetate (53.9%)	Lipase CAL-B (100.0 mg)			

**Table 2:** ^1^H chemical shift (δ) of resveratrol, resveratrol monocaprylate.

^1^H Position	Resveratrol	R-4′-caprylate	R-3-caprylate	R-3,5,4′-tricaprylate
2	6.40	6.40	6.75	6.96
4	6.12	6.12	6.39	6.78
6	6.40	6.40	6.68	6.96
7	6.82	Overlapping	6.88	7.03
8	6.95	Overlapping	7.04	7.09
2′	7.40	7.55	7.36	7.45
3′	6.77	7.05	6.72	7.06
5′	6.77	7.05	6.72	7.06
6′	7.40	7.55	7.36	7.45
3-OH	9.22	9.20	-	-
5-OH	9.22	9.20	9.53	-
4′-OH	9.57	-	9.63	-

**Table 3:** Lipophilicity and MW (molecular weight) of resveratrol esters ^a^.

Monoesters, MW (Da)	Log P (4′-acylation)	Log P (3-acylation)	Diesters, MW (Da)	Log P (4′,3 or 3,5-acylation)	Triesters, MW (Da)	Log P (3,5,4′-acylation)
-monoacetate, 270	3.81	3.78	-diacetate, 312	4.16	-triacetate, 354	3.75
-monobutyrate, 298	4.62	4.55	-dibutyrate, 368	5.48	-tributyrate, 438	6.03
-monocaproate, 326	5.40	5.31	-dicaproate, 424	6.77	-tricaproate, 522	7.50
-monocaprylate, 354	5.88	5.85	-dicaprylate,480	8.27	-triocaprylate,606	9.29
-moncaprate, 382	6.53	6.45	-dicaprate, 536	9.31	-tricaprate, 690	10.13
-monolaurate, 410	7.34	7.28	-dilaurate, 592	10.01	-trilaurate, 774	10.64
-monomyristate, 438	8.15	8.06	-dimyristate,648	10.49	-trimyristate, 858	10.86
-monostearate, 494	9.34	9.30	-distearate, 760	11.05	-tristearate, 1026	10.90

^a^ The lipophilicity of resveratrol derivatives was calculated using ALOGPS 2.1. The structures of resveratrol derivatives in the SMILE system were drawn by ChemBioDraw Ultra 12.0.

## Data Availability

Not applicable.

## Supplementary Materials

Figure S1: MS results of resveratrol monolaurate in negative mode, Figure S2: Original result of ^1^H-NMR of resveratrol, Figure S3: Original result of ^1^H-NMR of resveratrol monocaprylate, Figure S4: Original result of ^1^H-NMR of resveratrol monocaprate, Figure S5: Original result of ^1^H-NMR of resveratrol monocaproate, Figure S6: Original result of ^1^H-NMR of resveratrol monobutyrate, Figure S7: Original result of ^1^H-NMR of partially purified resveratrol tricaprylate.

## Sample Availability

Samples of the compounds are available from the authors.
