# Supplementary material for: The Effects of Acyl Chain Length on Antioxidant Efficacy of Mono- and Multi-Acylated Resveratrol: A Comparative Assessment
Source: Molecules. 2022 Feb 2;27(3):1001. doi: 10.3390/molecules27031001 (PMC8839368; doi:10.3390/molecules27031001)

Figure S1. MS results of resveratrol monolaurate in negative mode.

A. Original result of MS of mixture containing monolaurate and dilaurate resveratrol.

B. Original result of MS of purified monolaurate resveratrol.

Figure S2. Original result of ^{1}H-NMR of resveratrol.

Figure S3. Original result of ^{1}H-NMR of resveratrol monocaprylate.

Figure S4. Original result of ^{1}H-NMR of resveratrol monocaprate.

Figure S5. Original result of ^{1}H-NMR of resveratrol monocaproate.

Figure S6. Original result of ^{1}H-NMR of resveratrol monobutyrate.

Figure S7. Original result of ^{1}H-NMR of partially purified resveratrol tricaprylate.

Figure S1. Mass spectra of resveratrol monolaurate (before and after purification).

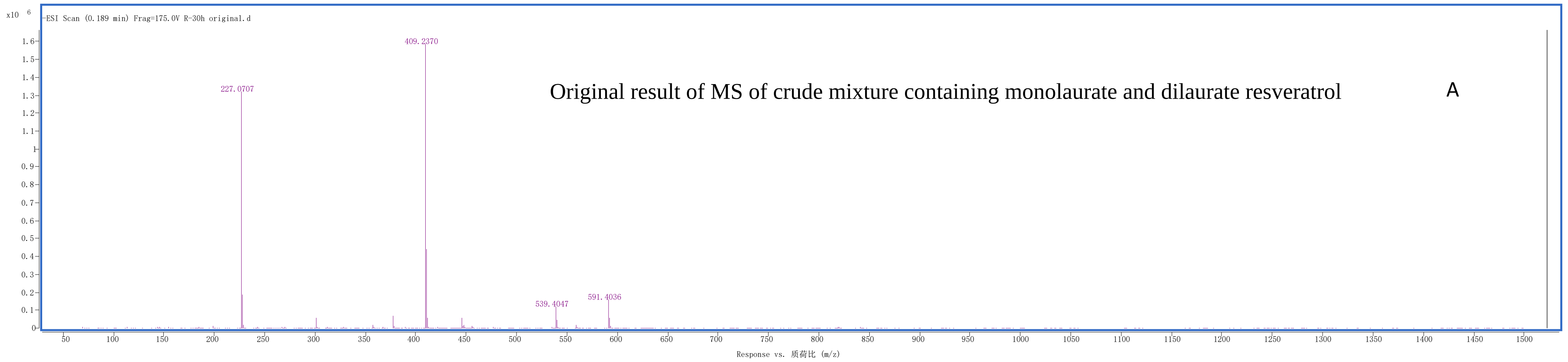

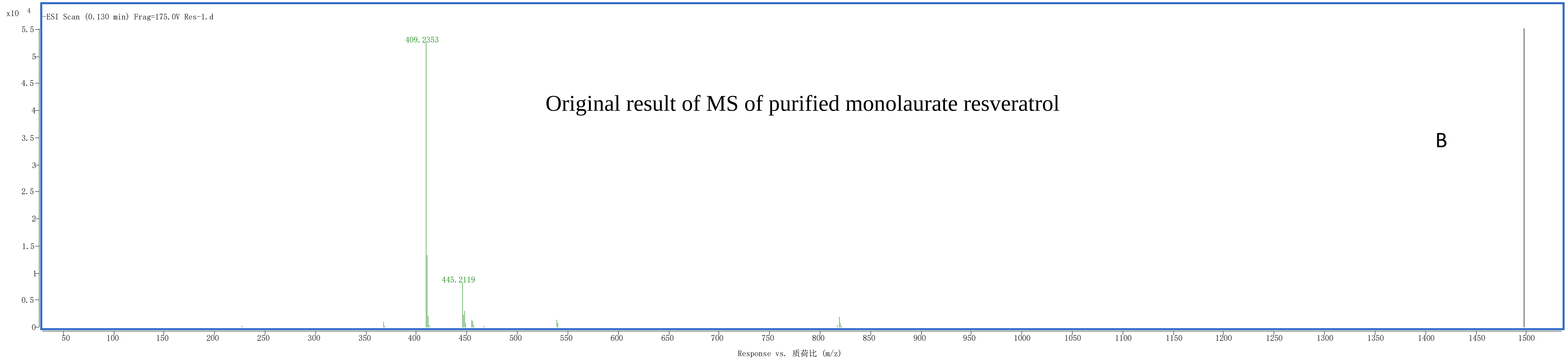

Figure S2. ^{1}H-NMR of resveratrol.

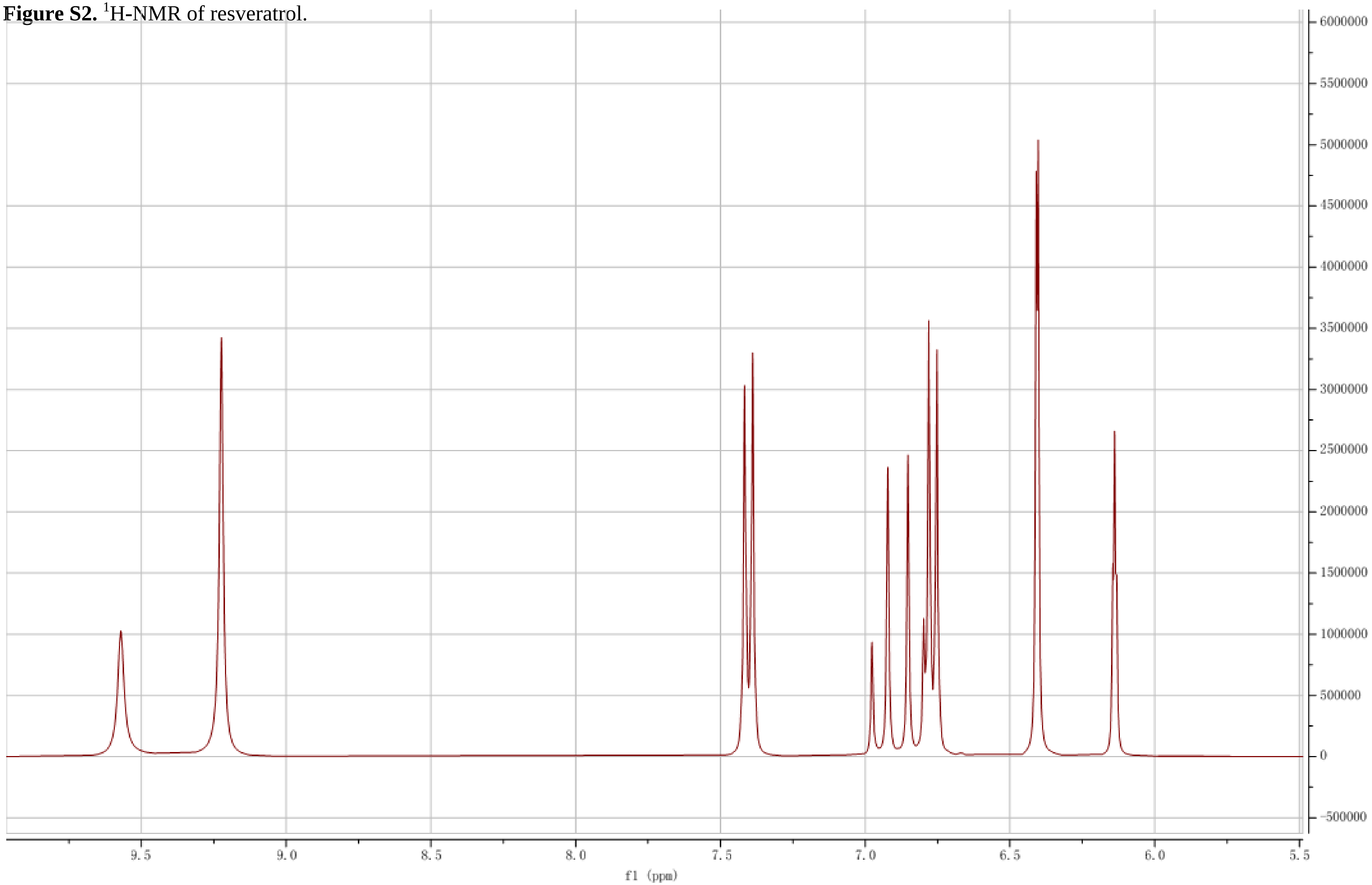

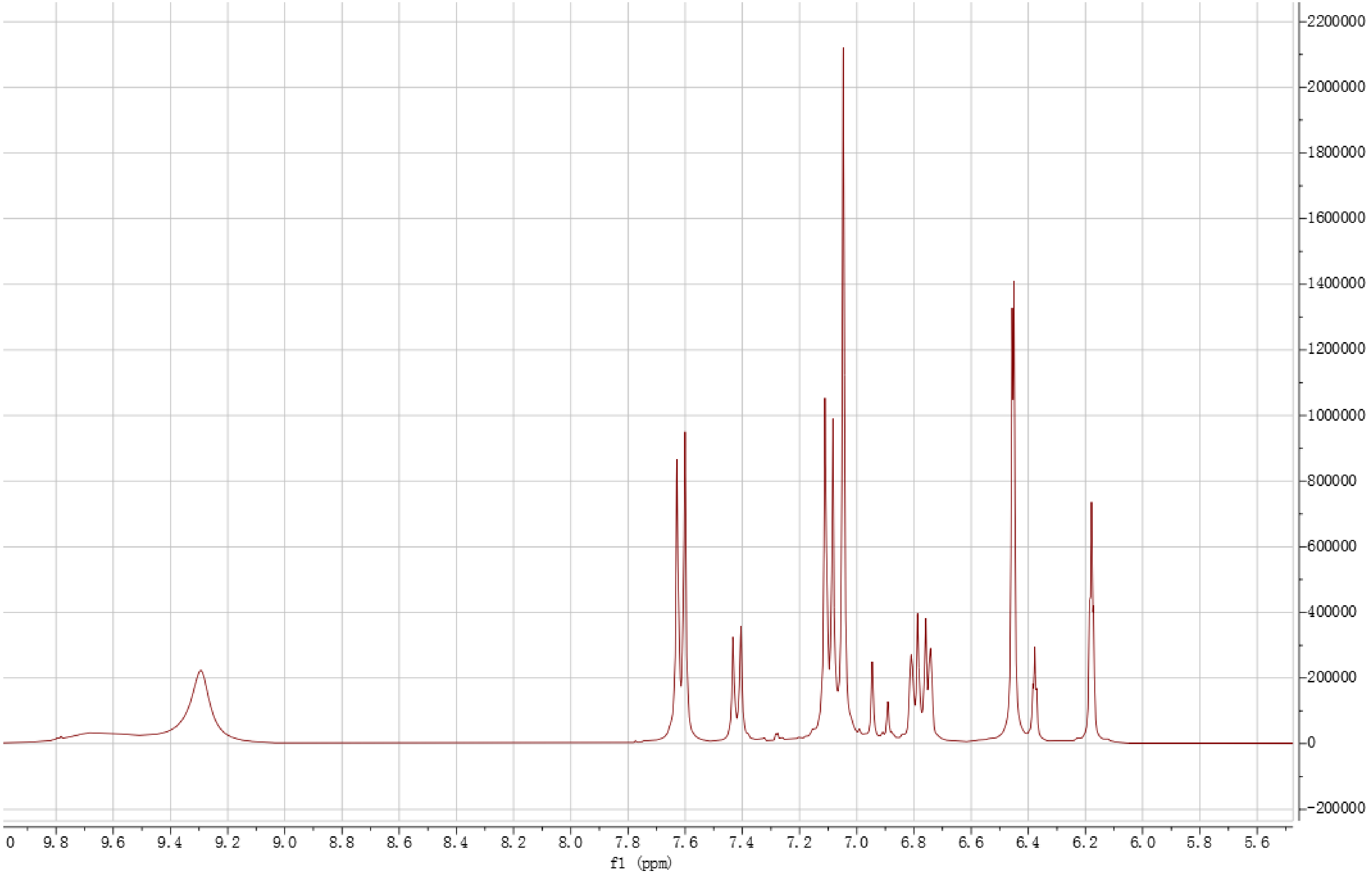

Figure S3. ^{1}H-NMR of resveratrol monocaprylate.

Figure S4. ^{1}H-NMR of resveratrol monocaprate.

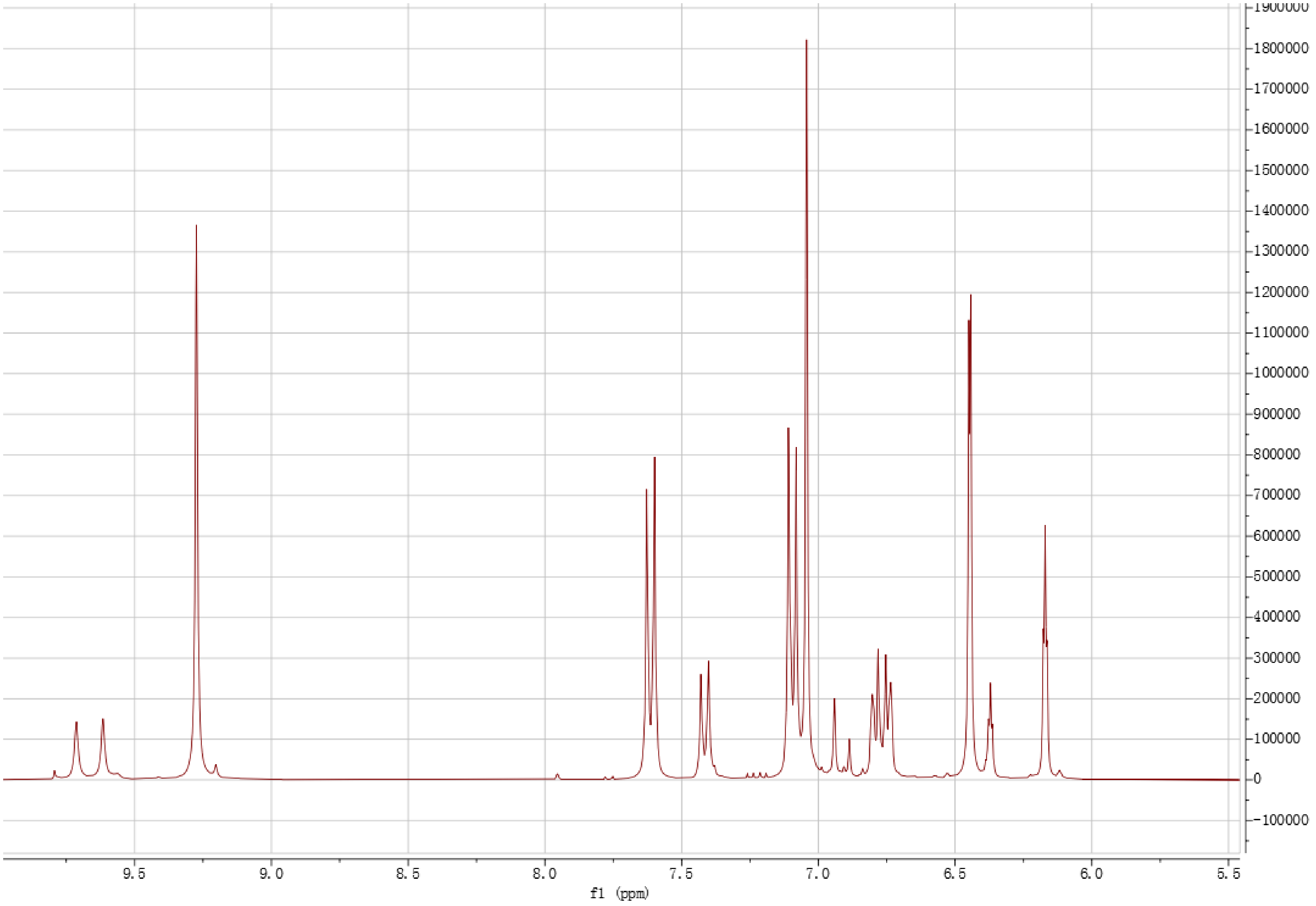

Figure S5. ^{1}H-NMR of resveratrol monocaproate.

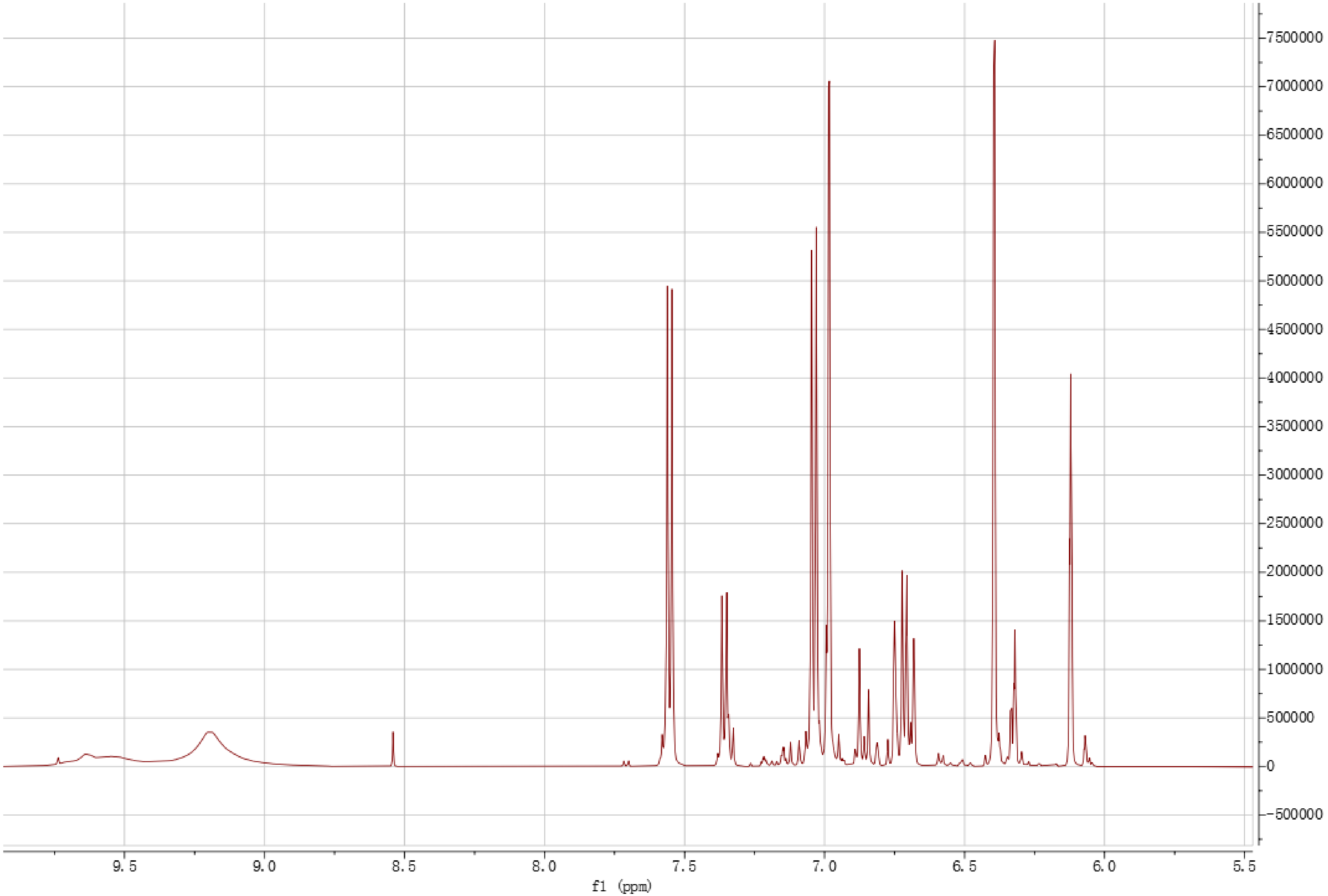

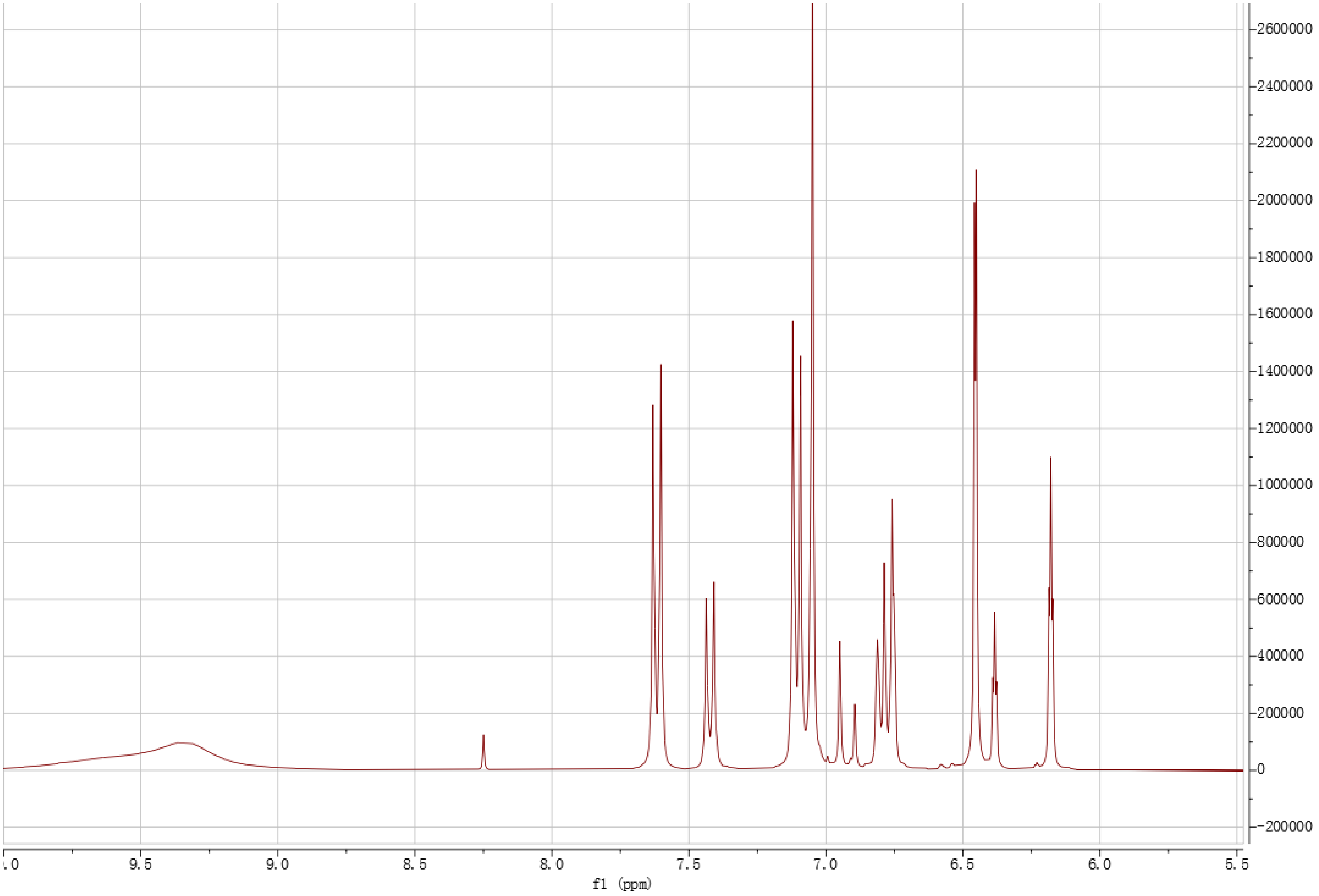

Figure S6. ^{1}H-NMR of resveratrol monobutyrate.

Figure S7. ^{1}H-NMR of resveratrol tricaprylate.

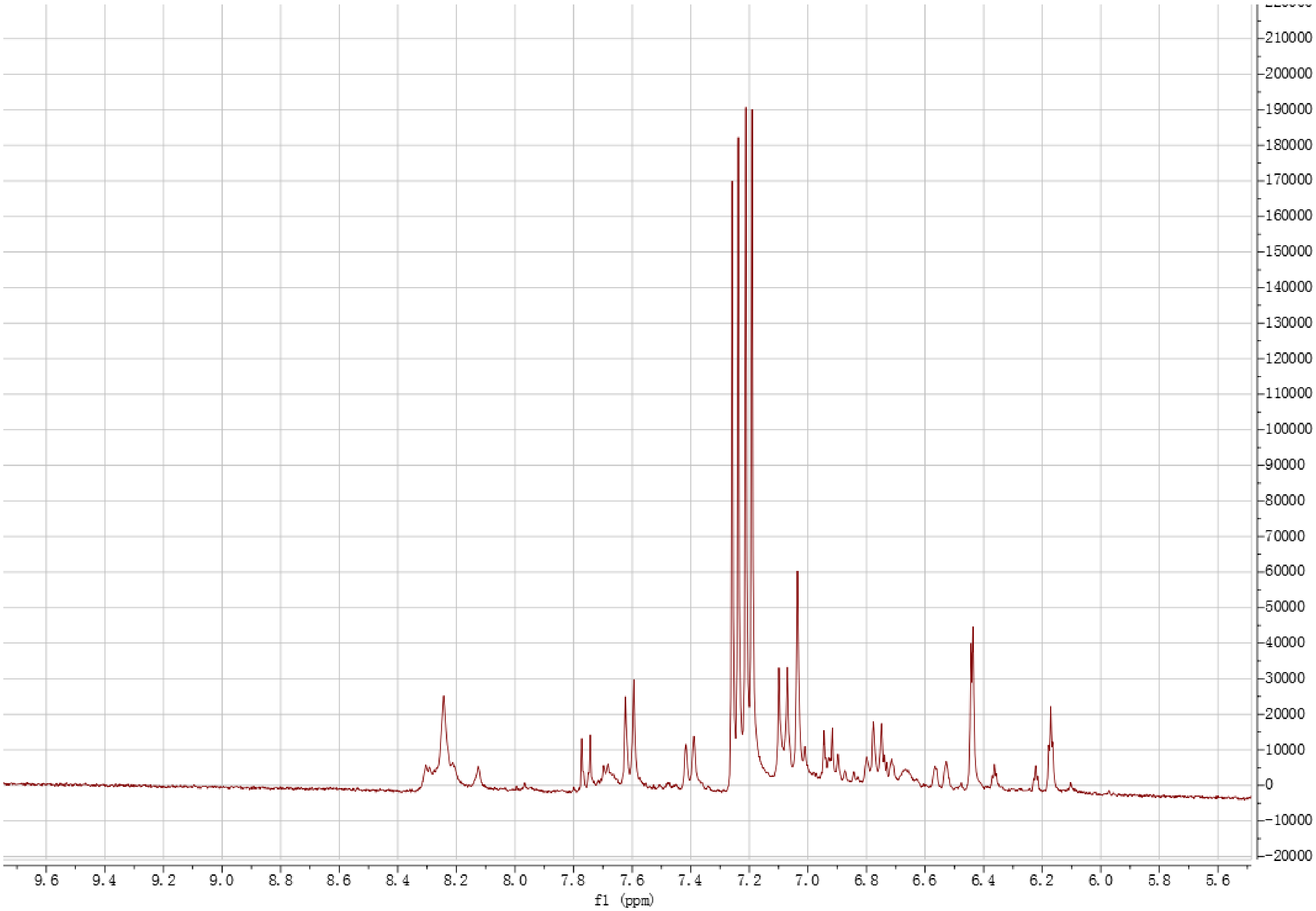

Supplement: Supplementary file 1 [file molecules-27-01001-s001.zip › molecules-1532505.pdf]
